# Using a Medical Intranet of Things System to Prevent Bed Falls in an Acute Care Hospital: A Pilot Study

**DOI:** 10.2196/jmir.7131

**Published:** 2017-05-04

**Authors:** Henri U Balaguera, Diana Wise, Chun Yin Ng, Han-Wen Tso, Wan-Lin Chiang, Aimee M Hutchinson, Tracy Galvin, Lee Hilborne, Cathy Hoffman, Chi-Cheng Huang, C Jason Wang

**Affiliations:** ^1^ Lahey Hospital and Medical Center Burlington, MA United States; ^2^ Tufts University School of Medicine Boston, MA United States; ^3^ MedicusTek USA Corporation Newport Beach, CA United States; ^4^ MedicusTek, Inc Taipei Taiwan; ^5^ David Geffen School of Medicine at UCLA Department of Pathology and Laboratory Medicine Los Angeles, CA United States; ^6^ Stanford University School of Medicine Stanford, CA United States

**Keywords:** accidental falls, acute care, nursing, patient safety, patient-centered care, sensor devices and platforms, health care technology, mobile apps, patient monitoring, health technology assessment

## Abstract

**Background:**

Hospitalized patients in the United States experience falls at a rate of 2.6 to 17.1 per 1000 patient-days, with the majority occurring when a patient is moving to, from, and around the bed. Each fall with injury costs an average of US $14,000.

**Objective:**

The aim was to conduct a technology evaluation, including feasibility, usability, and user experience, of a medical sensor-based Intranet of things (IoT) system in facilitating nursing response to bed exits in an acute care hospital.

**Methods:**

Patients 18 years and older with a Morse fall score of 45 or greater were recruited from a 35-bed medical-surgical ward in a 317-bed Massachusetts teaching hospital. Eligible patients were recruited between August 4, 2015 and July 31, 2016. Participants received a sensor pad placed between the top of their mattress and bed sheet. The sensor pad was positioned to monitor movement from patients’ shoulders to their thighs. The SensableCare System was evaluated for monitoring patient movement and delivering timely alerts to nursing staff via mobile devices when there appeared to be a bed-exit attempt. Sensor pad data were collected automatically from the system. The primary outcomes included number of falls, time to turn off bed-exit alerts, and the number of attempted bed-exit events. Data on patient falls were collected by clinical research assistants and confirmed with the unit nurse manager. Explanatory variables included room locations (zones 1-3), day of the week, nursing shift, and Morse Fall Scale (ie, positive fall history, positive secondary diagnosis, positive ambulatory aid, weak impaired gait/transfer, positive IV/saline lock, mentally forgets limitations). We also assessed user experience via nurse focus groups. Qualitative data regarding staff interactions with the system were collected during two focus groups with 25 total nurses, each lasting approximately 1.5 hours.

**Results:**

A total of 91 patients used the system for 234.0 patient-days and experienced no bed falls during the study period. On average, patients were assisted/returned to bed 46 seconds after the alert system was triggered. Response times were longer during the overnight nursing shift versus day shift (*P*=.005), but were independent of the patient’s location on the unit. Focus groups revealed that nurses found the system integrated well into the clinical nursing workflow and the alerts were helpful in patient monitoring.

**Conclusions:**

A medical IoT system can be integrated into the existing nursing workflow and may reduce patient bed fall risk in acute care hospitals, a high priority but an elusive patient safety challenge. By using an alerting system that sends notifications directly to nurses’ mobile devices, nurses can equally respond to unassisted bed-exit attempts wherever patients are located on the ward. Further study, including a fully powered randomized controlled trial, is needed to assess effectiveness across hospital settings.

## Introduction

Each year in the United States, between 700,000 and 1,000,000 hospitalized patients fall [1]. Studies report fall rates between 2.6 and 17.1 per 1000 patient-days with 30% to 50% resulting in injury [2-6]. Several observational studies report that 60% to 80% of in-hospital falls occur when a patient is moving to, from, and around the bed, and 80% of falls are unassisted [7-9]. Cost analysis studies estimate inpatient falls add approximately US $3500 to US $14,000 per patient stay, depending on whether there is serious injury [5,10]. The US Centers for Medicare and Medicaid Services (CMS) lists falls as one of 14 categories of preventable hospital-acquired conditions. Since 2008, CMS no longer pays for the extra health care costs associated with hospital-acquired falls, shifting the cost burden to hospitals [2,11,12].

Fall prevention strategies using innovative medical technology can potentially improve health care delivery and patient safety [13]. A range of medical-alerting devices have already begun using wireless sensor technologies to target pressure injuries and vital sign monitoring; however, only a few have published their results [14-15].

Although alerting technology can help nurses, inaccurate or false alarms can have the opposite effect on both caregivers and patients. Studies show 80% to 99% of monitor alarms are false or clinically insignificant [16-18]. With low positive predictive value (PPV) for monitoring alerts, nurses may experience alarm fatigue. Repeated false alarms leads to desensitization and true alarms requiring intervention could be ignored [19]. An alert monitoring system with a high PPV, routed to the appropriate caregiver or clinician, is needed to provide alerts that will lead to timely action. Furthermore, increasing alert PPV could minimize sleep disruption and positively affect recovery for patients [20].

**Figure 1:**
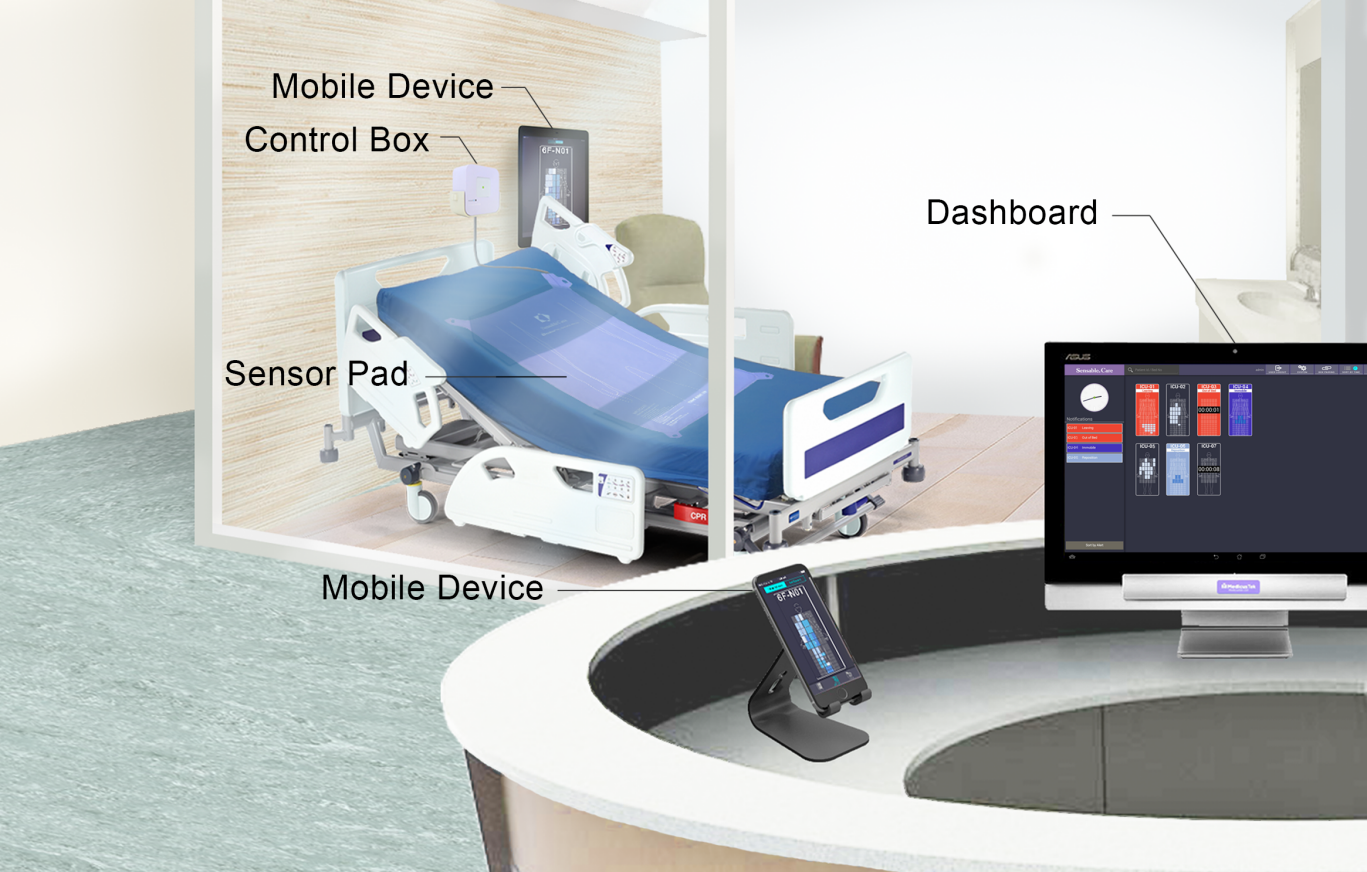
Components of the SensableCare System.

We report the use of a medical Intranet of things (IoT) system to assess how quickly nurses respond to bed exits in an acute care hospital setting where an automated bed sensor pad system was used to analyze real-time patient movement data and provide timely alerts to nursing staff, with the potential to ultimately reduce bed falls (see Figure 1 for system setup in the hospital setting).

## Methods

### Setting

This study was conducted at a 317-bed suburban Massachusetts teaching hospital for 12 months from August 4, 2015 to July 31, 2016. Patients were recruited from a 35-bed medical-surgical ward. Patients 18 years and older and deemed a high fall risk (Morse Fall Scale score ≥45) were eligible for inclusion in the study [21]. Vulnerable populations (eg, prisoners, patients undergoing stem cell transplant) were excluded. Patients were placed throughout the ward; high fall risk patients (eg, those with encephalopathy) were generally placed in rooms in zone 1 (within 40 feet of the nursing station). Zones 2 and 3 rooms were 41 to 85 feet and 86 to 110 feet away from the nursing station, respectively.

#### Patient Consent

**Figure 2:**
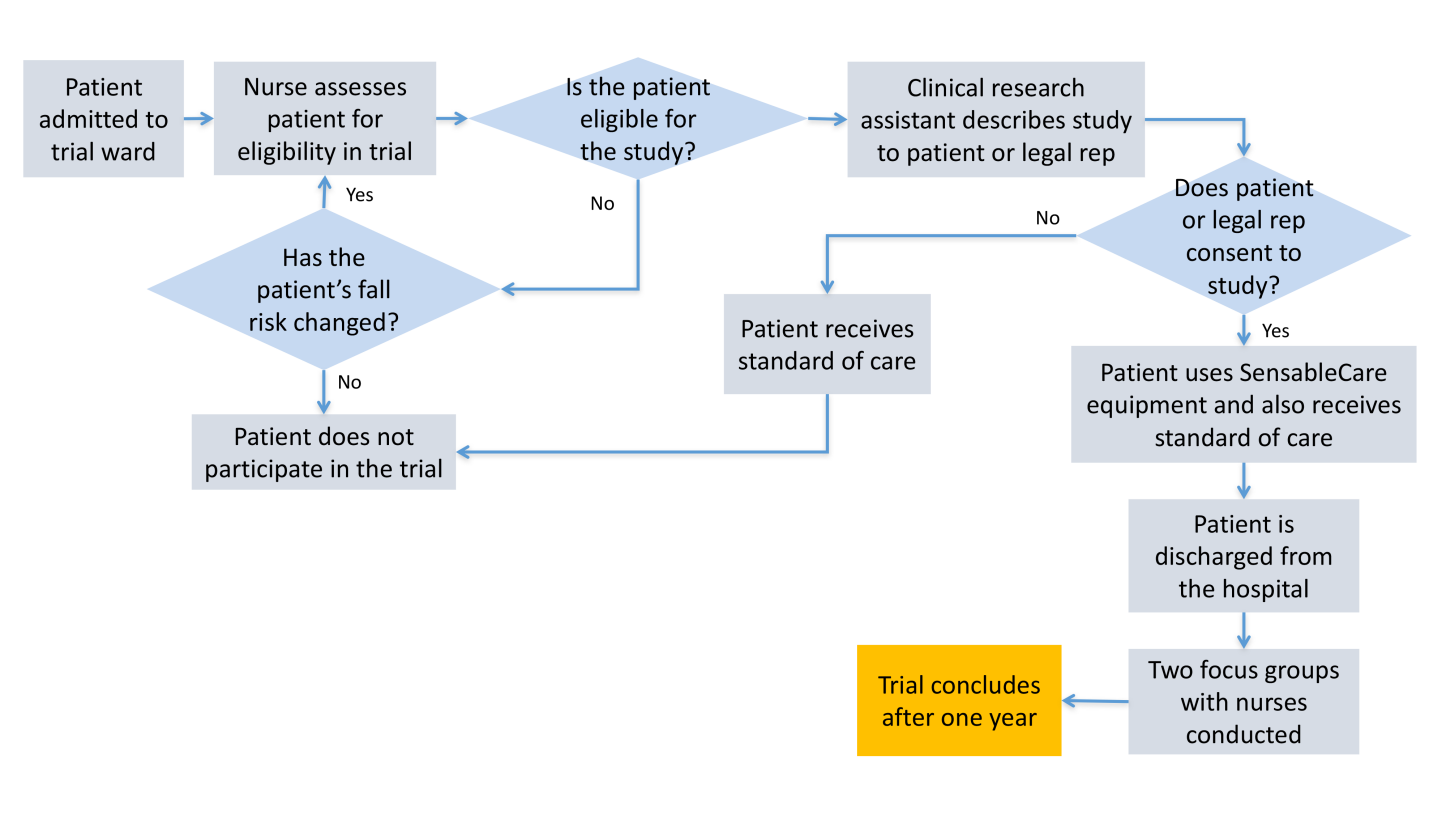
Recruitment methodology.

Clinical research assistants talked with eligible patients about the study when they were first admitted to the ward. Patients interested in participating signed a written consent form. For patients who could not readily consent on their own, family members and legal representatives could consent on behalf of the patient (see Figure 2). Basic demographic information was collected from eligible patients along with their Morse Fall Scale to confirm eligibility. This study was approved by the Lahey Hospital and Medical Center Institutional Review Board (IRB).

#### Medical Intranet of Things System Setup

**Figure 3:**
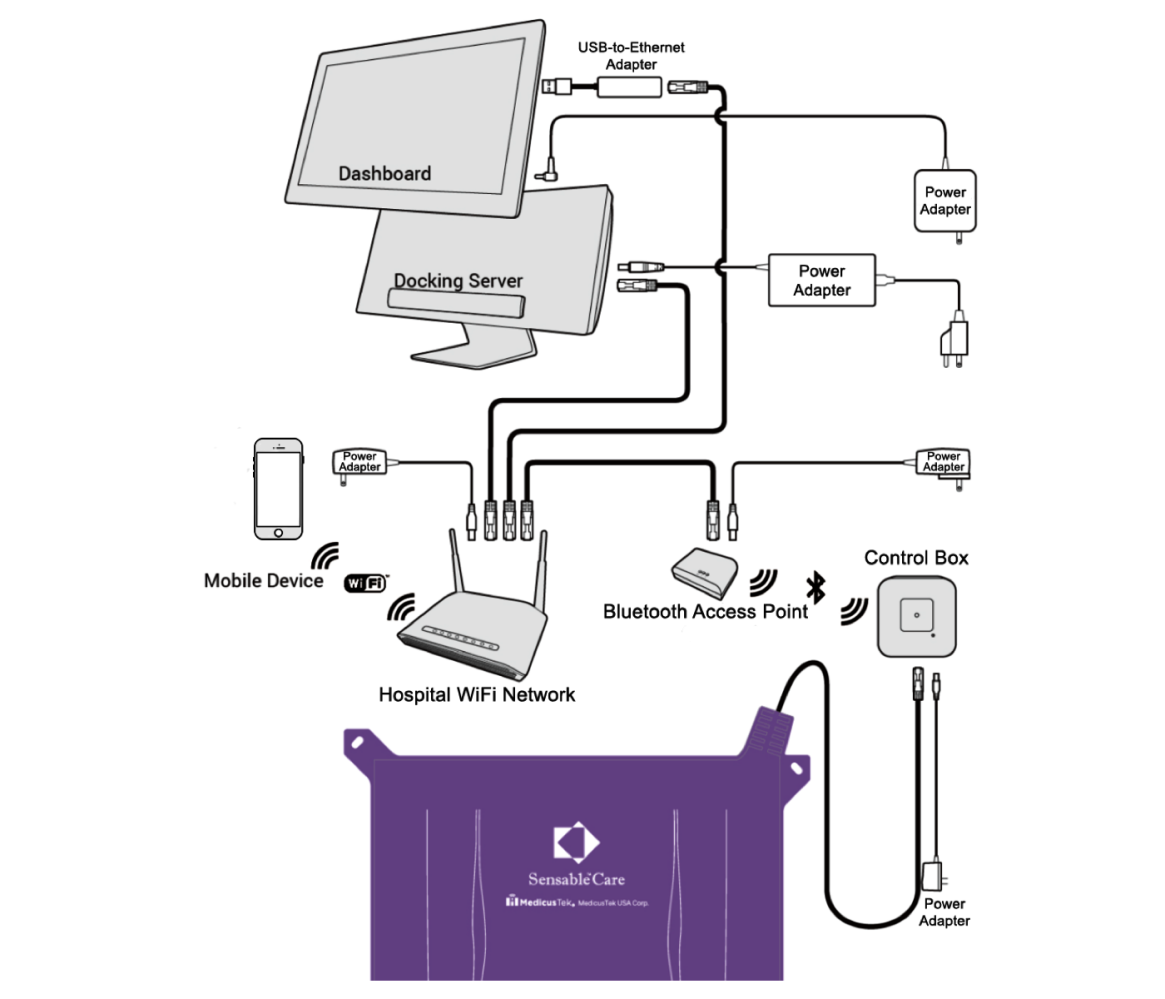
Intranet of things architecture of the SensableCare System. The sensor pad sends data through a cable to the control box located at the patient’s bedside. The control box wirelessly transmits this data to the Bluetooth routers located throughout the ward. This information then travels through the hospital network to the dashboard and docking server where the data is analyzed. When an alert is sent to the nurse via an app on their mobile phone, it is wirelessly transmitted through the hospital’s Wi-Fi network.

The study tested the SensableCare System for reducing bed falls. A pressure-sensing pad was placed between the mattress surface and the bed sheet (Figure 3). The sensor pad was positioned so it monitored movement from the patients’ shoulder to thighs. The pad is approximately 46 inches by 30 inches and encased in a waterproof nylon cover that is coated to provide bacterial resistance. It covered 49.3% of the bed and reached 2.5 inches from the edge of the bed. The pad sensor array detects when pressure is applied. When the system recognizes an increased probability of a bed exit, the system’s software algorithm alerts the nursing staff via an app on a mobile device (ie, mobile phone) that nurses carry and to a dashboard at the nursing station. When the patient attempts to leave the bed, an audible message comes from a control box next to the patient’s bed reminding the patient not to leave and that a nurse will arrive shortly. Concurrently, nurses receive the alerts at a dashboard and through their mobile phone via audible, vibratory, and visual alerts to prompt them for rapid response. All the system components are connected to the Intranet, allowing caregivers to receive actionable alerts whenever their mobile device is online. Each device on the network is identified by its IP address or MAC address.

The SensableCare System allows nurses to visualize how the patient is positioned in bed at the time of the alert, from which room number the alert is being generated, and informs other nurses when a patient is being assisted. Using a predictive algorithm, the system can also identify patients’ activities in bed (eg, whether a patient begins to stir in bed, sits up in bed), before the patient attempts to leave the bed, or is already out of bed.

Nurses are able to customize each individual patient’s alert settings. Nurses receive bed-leaving alerts when the system detects the patient attempting to leave the bed or is out of the bed. Nurses can also choose to receive alerts earlier, when a patient is stirring after being still for longer than 20 minutes and/or when a patient is sitting up in bed. Both the nurse and nurse aide responsible for an enrolled patient would receive alerts directly through their mobile phones via a real-time push notification. The charge nurse at the nursing station would also receive alerts through the SensableCare app on the dashboard. Nurses respond to the alert by tapping the control box in the patient’s room or returning the patient back to bed, in order to turn off the alerts at the dashboard and on other nurses’ mobile phones (ie, Apple iPhone for this trial).

As a condition for the trial, the hospital’s existing bed alarm (Stryker Secure II hospital bed with a bed-exit system) was maintained and used concurrently with the SensableCare System for patients who consented to the study. Those who did not consent to the study only used the existing Stryker bed alarm. The Stryker bed signals when a patient is exiting the bed via an audible alert. A high-pitched sound signifies the patient is leaving or is out of bed. A hallway light outside the patient room flashes in conjunction with the audible alert. The nurses would listen for the sound and go to the room with the flashing hallway light to assist the patient. Patients not participating in the study continued to only use the hospital’s existing bed fall alarms.

#### Bed Fall Alerts and Patient Positioning Data

Sensor pad data were collected automatically from the system. These data included patient positioning data, the times the alert was sent to nursing staff, time to turn off bed-exit alerts, when a patient returned to bed, and the duration a patient spent out of bed. Data on patient falls were collected by the clinical research assistants and confirmed with the nurse manager of the unit.

In a previous engineering validation study of 47 patients in a hospital using a low-resolution camera to match movement captured with the sensor pad data, the PPV was 78.5% (unpublished data). System specificity is difficult to calculate in this system because it refers to the true negative rate in which a patient did not leave the bed and the system did not generate an alert. In this study, we calculated the PPV as it was implemented at Lahey Hospital and Medical Center. Because this was a 1-year pilot study approved to understand the integration of medical technology into hospital workflow, sample size calculation was not performed prior to the study. However, the fall rates using the SensableCare System can be compared to the study unit’s historical fall rate with the existing bed alarm during a 12-month period before the trial. The historical unit fall rate during the 12-month period prior to the study was 2.4 falls per 1000 patient-days.

### Quantitative Data Analysis

#### Independent and Dependent Variables

The primary dependent variable of this study was nurse response time to alerts. Time-to-response data were calculated for all events when a nurse responded to an alert within 5 minutes; alarms turned off after 5 minutes were excluded because they were observed to be situations in which patients were assisted without the nurse first disabling the alarm.

To assess possible factors that may predict nurses’ response times to alerts, this study included the following independent variables: demographic characteristics of patients (sex, age, weight, and fall risk factors) and characteristics of alerts (alert room, alert day, and alert time). Alert room was categorized as follows: zone 1, which is closest to the nursing station (within 40 feet from nursing station); zone 2, rooms further from the nursing station (41-85 feet away); and zone 3, rooms furthest from nursing station (86-110 feet away). Alert day was classified as “weekday” or “weekend.” Alert time was divided into three groups: day shift, evening shift, and overnight shift.

We conducted descriptive analyses to provide a sociodemographic profile of patients and the correlations of alert characteristics and nurses’ response times to alerts. After descriptive analysis, linear regression with generalized estimating equations (GEE) was used to examine the relationship among various factors and the alert response time. All *P* values were two-sided. Analyses were done using SAS version 9.4 (SAS Institute Inc, Cary, NC, USA).

### Qualitative Study With Focus Groups

Qualitative data regarding staff interactions with the system were collected during two focus group sessions with a total of 25 nurses, each lasting approximately 1.5 hours. The focus groups were held on consecutive days to allow nurses separate opportunities to attend. Two research staff conducted the focus group with one serving as moderator and the other as note taker. Nurse managers invited all hospital staff working in the ward to voluntarily participate without monetary compensation. Research staff used a prepared list of questions approved by the IRB to facilitate focus group discussion. Detailed transcriptions were analyzed for themes related to barriers and facilitators for integrating the system into nurses’ clinical workflow.

## Results

### Falls and Response to Alerts

During the study period, 91 patients used the system for 234.0 patient-days and experienced no bed falls. Both male and female patients with a range of ages, weights, and Morse fall scores participated in the trial (Table 1).

**Table 1:** Patient profiles (N=91).

Characteristics			Participants	
**Sex, n (%)**				
	Male		44 (48)	
	Female		47 (52)	
**Age (years), n (%)**				
	<65		20 (22)	
	65-74		22 (24)	
	≥75		49 (54)	
**Weight (lb), n (%)**				
	<150		24 (26)	
	150-199		37 (41)	
	≥200		26 (29)	
	Missing^a^		4 (4)	
**Morse Fall Score, n (%)**				
	45-60		41 (45)	
	65-80		20 (22)	
	85-125		30 (33)	
**Zone,^b^n (%)**				
	Zone 1		29 (32)	
	Zone 2		29 (32)	
	Zone 3		33 (36)	
**Patient-days on trial**				
	Total, n		234.0	
	Per patient, mean (SD)		2.6 (2.1)	
**Morse Fall Score items (Morse Fall Score points), n (%)**				
	**Falls history**			
		No (0)		39 (43)
		Yes (25)		52 (57)
	**Secondary diagnosis**			
		No (0)		2 (2)
		Yes (15)		89 (98)
	**Ambulatory aid**			
		Bed rest/nurse assist (0)		43 (47)
		Crutches/cane/walker (15)		48 (53)
		Furniture (30)		0 (0)
	**IV/saline lock**			
		No (0)		2 (2)
		Yes (20)		89 (98)
	**Gait/Transferring**			
		Normal/bedrest/immobile (0)		13 (14)
		Weak (10)		74 (81)
		Impaired (20)		4 (4)
	**Mental status**			
		Oriented to own ability (0)		74 (81)
		Forgets limitations (15)		17 (19)

^a^ Weights were not recorded in the electronic health record.

^b^ Zone 1: closest to nursing station (within 40 feet from nursing station); zone 2: rooms further from nurse station (41-85 feet from nursing station); zone 3: rooms furthest from nursing station (86-110 feet from nursing station).

Nursing staff responded to alerts on average within a mean 45.9 (SD 64.7) seconds (Table 2). The SensableCare System bed alarm’s PPV was 62.1% (260 positive bed-leaving attempts/419 total bed alerts). The alert reset times among the three shifts were mean 42.3 (SD 62.4) seconds for day shift; mean 43.7 (SD 61.2) seconds for night shift, and mean 57.1 (SD 74.0) seconds for overnight shift; the overnight shift required more time to respond to alerts (*P*=.004; see Table 2).

**Table 2:** Alert characteristics and response.

Alert characteristics		Events, n	Alerts reset (within 5 min),^a^ n	Time to turn off alert (sec), mean (SD)	*P*^b^
Total events (bed leaving/out of bed)		1645	1416	45.9 (64.7)	
**Alert room^c^**					.002
	Zone 1	710	628	41.3 (58.3)	
	Zone 2	413	363	42.8 (61.1)	
	Zone 3	522	425	55.2 (75.0)	
**Alert day**					.17
	Weekday	1200	1040	47.2 (66.3)	
	Weekend	445	376	42.1 (59.9)	
**Alert time^d^(by shift)**					.004
	Day shift	703	581	42.3 (62.4)	
	Night shift	612	543	43.7 (61.2)	
	Overnight shift	330	292	57.1 (74.0)	

^a^ If alert was reset or patient return to bed was >5 minutes, they were considered unaddressed alerts and not included in this table.

^b^ Using *t* test (two groups) or ANOVA (three groups).

^c^ Zone1: within 40 feet of nursing station; zone2: within 40-85 feet of nursing station; zone 3: within 85-110 feet of nursing station.

^d^ Day shift: 07:00-14:59; evening shift: 15:00-22:59; overnight shift: 23:00-06:59 #

In multivariate linear regression using GEE (Table 3), response times were longer during the overnight nursing shift (beta=14.22, *P*=.005) compared to the day shift. Patient characteristics, alert room, and alert day were not significantly associated with alert response time.

**Table 3:** Factors predicting response time: multivariate linear regression using GEE.

Response time factors				Beta (SE)		*P*
**Patient characteristics**						
	**Sex (ref: female)**					
		Male	–5.07 (6.44)		.43	
	**Age, year (ref: <65)**					
		65-74	7.58 (10.10)		.45	
		≥75	6.63 (8.34)		.43	
	**Weight, lb (ref: 150-199)**					
		<150	–11.88 (6.45)		.07	
		≥200	13.01 (9.52)		.17	
	**Falls history (ref: no)**					
		Yes	8.93 (5.07)		.08	
	**Ambulatory aid (ref: bed rest/nurse assist)**					
		Crutches/cane/walker	–4.69 (5.39)		.38	
	**Gait/Transferring (ref: normal/bedrest/immobile)**					
		Weak/impaired	–5.05 (8.79)		.57	
	**Mental status (ref: oriented to own ability)**					
		Forgets limitations	–1.77 (5.94)		.77	
**Alert characteristics**						
	**Alert room (ref: zone 2+3)**					
		Zone 1	–0.62 (4.76)		.90	
	**Alert day (ref: weekend)**					
		Weekday	0.99 (3.84)		.80	
	**Alert time (ref: day shift)**					
		Evening shift	0.65 (4.00)		.87	
		Overnight shift	14.22 (5.08)		.005	

#### User Experience

Focus group participants found the SensableCare System easy to use and valued it as an effective technology for reducing bed falls: “It is helpful for really weak and unsteady patients,” reported one participant. Participants were satisfied overall with the user interface, system design, and mobile phone notifications: “...instead of browsing, you can see [in] which room [the alarm] is going off...notifications on the phone instead of browsing through the hallway...I find it positive, takes you less time.” Specific alerts on patient statuses (ie, stirring, sitting up, leaving, out of bed) were informative in understanding patient behaviors and comfort levels: “I like to know that [the patient] has moved in bed, my patient attempted to get out of bed, etc” and “Stirring shows patient is a little uncomfortable in the bed so check on them anyway.” There were self-reported instances in which nurses did not respond to an alert (ie, nurses were not carrying their mobile phones, when they were caring for another patient, or when they were busy and otherwise unable to check the phone): “Sometimes I can’t drop what I am doing to check the phone.” Nurses generally found the current SensableCare System useful and integrated well into their nursing workflow. Participants expressed that they could envision the system in multiple hospital settings.

When there were false alerts, nurses expressed that the system sometimes would sound when the patient was going back to bed. This was quickly remedied by tapping the control box when the nurse was at the patient bedside. Patients who rolled over toward the edge of the bed could also trigger a leaving alert. However, in those instances, nurses expressed that it was a minor inconvenience to have the ability to prevent bed falls for their patients.

## Discussion

To our knowledge, this is the first study using a predictive algorithm with data collected from sensors to proactively reduce the fall risk in an acute care setting. By providing nurses with alerts to unassisted bed exits, the study looked at whether nurses responded quickly using this system. The ability for such a bed alert system to help prevent unassisted bed-leaving events might depend on at least three key technology factors: increased time for nursing/caregiver response, enabling nurses to respond from wherever they are, and eliciting a specific response to prevent bed falls.

On average, nurses reset the alarm approximately 46 seconds after an alert was triggered. Because the system’s software algorithm enables an alert to nursing staff’s mobile device directly when a patient is initiating a departure from bed, they have more time to respond, hence decreasing the likelihood of bed falls. Furthermore, nurses can choose to receive earlier notifications (eg, patients stirring, sitting up), creating a graduated sequence of potential bed-exit notifications. Giving nurses additional time to respond to a potential bed exit allows them to respond in an appropriate and timely manner. Because nurses must balance multiple patient care responsibilities, it may take some time to drop what they are currently doing to address an immediate patient safety risk. Furthermore, once nurses arrive at the bedside, they are likely to assist the patient first before turning off the sounding alarm. This sequence of events may increase the observed response time recorded by our system.

Currently, high fall risk patients are placed in zones closest to the nursing station for better monitoring and responsiveness to bed-leaving events. Our study found there is no significant correlation between a patient’s location on the ward and nursing response time. This may suggest that by using an alerting system that sends notifications directly to nurses’ mobile devices, nurses can equally respond to unassisted bed exits wherever patients are located on the ward. Nurses may no longer have to move patients to a room closer to the nursing station.

The PPV with the SensableCare System in this study is 62.1%. One multicenter study found that alarms from a typical physiologic monitor have PPVs of 27% [22]; the same study saw only 5.9% of the monitor alarms led to a nurse’s response [22]. Alarm fatigue may cause health care professionals to lower the alarm volume, adjust alarm settings to a point that is unsafe for the patient, or even ignore or deactivate the alarm. One study found that desensitization to alarms and missing alarms have been attributed to patient deaths [23]. SensableCare System’s high PPV may have reduced concerns for false alarms.

A medical IoT system may provide information that is helpful in improving hospital operations. Comparing unit alert response times to fall rates may give administrators a metric for potentially reducing bed falls. Nurses can use the number of alerts generated during a particular shift to think about staffing levels and whether they are adequate to address potential bed fall events in a timely manner. Moreover, patient experience will improve with less noise on the ward when alerts are sent to specific nurses.

In summary, we have demonstrated that the SensableCare System is feasible and can be integrated to acute care hospitals. Nurses responded to the system bed alerts quickly and saw no falls for patients who participated during the pilot study. Further work to understand the total cost of ownership to operate a medical IoT can help hospital administrators calculate the cost benefits of using such a system. These costs can be determined if a hospital uses the SensableCare System to optimize nurse staffing or uses a dedicated staff member to carry a mobile device to quickly respond and help patients in a ward.

There are some limitations to this pilot study, including the small number of patients, recruitment of only high fall risk patients, and data from a single hospital unit. Moreover, the standard of care for preventing bed falls, which was the hospital’s existing bed alarm, was turned on while the study was taking place on the same beds. This could underestimate the effect of the system if used alone. Also, because nurses were aware that fall risk and the SensableCare System were being studied, they may have been more vigilant (ie, Hawthorne effect) when using the system.

In conclusion, the preliminary evidence suggests that a technological solution using IoT may mitigate the long-standing patient safety fall risk in acute care hospitals while providing hospitals with baseline information for quality improvement, including response time from alarm to assist. Further study is necessary to fully assess the effectiveness of such systems in hospital settings.

## Abbreviations

GEE: generalized estimating equation
IoT: Intranet of things
IRB: Institutional Review Board
PPV: positive predictive value
